# Synergistic Cytotoxicity of Nano-titanium Dioxide and Phthalocyanine on HepG2 Cells via Sonophotodynamic Therapy

**DOI:** 10.1007/s12011-025-04660-8

**Published:** 2025-05-14

**Authors:** Adem Yavaş, Ömer Kesmez, Feride Demir, Mehran Aksel

**Affiliations:** 1 Food Processing Department, Food Quality Control and Analysis Programme, Çine Vocational School, Aydin Adnan Menderes University, Aydin, 09500 Türkiye; 2 Agricultural Biotechnology and Food Safety Research and Application Center, Adnan Menderes University, Aydin, 09970 Türkiye; 3 Department of Chemistry, Faculty of Science, Akdeniz University, Antalya, 07058 Türkiye; 4 Department of Biophysics, Faculty of Medicine, Aydin Adnan Menderes University, Aydin, 09010 Türkiye

**Keywords:** Hepatocellular carcinoma, Sonophotodynamic therapy, Nano-titanium dioxide, Phthalocyanine, Apoptosis, Oxidative stress

## Abstract

Hepatocellular carcinoma (HCC) presents a significant therapeutic challenge, necessitating novel approaches beyond conventional treatments. This study investigates the combined cytotoxic effects of nano-titanium dioxide (nano-TiO₂), copper (II) phthalocyanine (CuPc), and copper (II) phthalocyanine–modified nano-TiO₂-(nano-TiO₂/Pc) on HepG2 hepatocellular carcinoma cells using sonodynamic therapy (SDT), photodynamic therapy (PDT), and sonophotodynamic therapy (SPDT). The results show that individual treatments with nano-TiO₂ or CuPc alone did not induce significant cytotoxicity. However, when combined with SDT or PDT, a noticeable decrease in cell viability was observed. Strikingly, SPDT combined with nano-TiO₂/Pc demonstrated the most significant cytotoxic effect, achieving up to 83.80% apoptosis in HepG2 cells. This was associated with a marked reduction in Bcl-2 protein levels and an increase in cleaved caspase-3, cleaved caspase-9, cytochrome-c (cyt-c), and Bax indicating the activation of both intrinsic and extrinsic apoptotic pathways. Furthermore, SPDT-nano-TiO₂/Pc significantly increased oxidative stress, as evidenced by decreased levels of superoxide dismutase (SOD), catalase (CAT), and glutathione (GSH), along with elevated levels of malondialdehyde (MDA). These findings suggest that phthalocyanine-mediated SPDT effectively enhances mitochondrial apoptosis and disrupts the tumor cytoplasmic membrane, highlighting the potential of combining SDT and PDT with nano-TiO₂/Pc as a promising strategy for cancer treatment.

**Graphical Abstract:**

**Figure:**
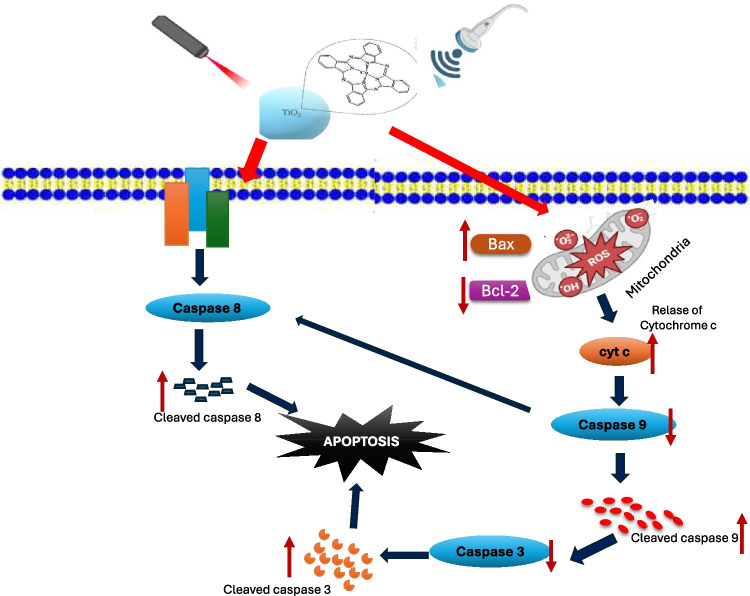
Schematic representation for the effect of nano-TiO_2_/Pc combined with SPDT on the intrinsic and extrinsic mitochondrial apoptotic pathway

## Introduction

Hepatocellular carcinoma (HCC) poses a considerable challenge to global health, with its rising incidence attributable to various contributors like environmental pollutants, detrimental lifestyle choices, and chronic stressors [1, 2]. The treatment of HCC typically requires a multifaceted approach, encompassing surgical intervention, radiation therapy, and drug-based therapies. However, this approach is fraught with difficulties, such as the potential for drug resistance, significant adverse effects, and substantial financial burdens [3, 4]. Therefore, there is a pressing need for innovative therapeutic options that can alleviate negative outcomes, improve treatment efficacy, and lessen the overall impact on patients. 

PDT and SDT represent innovative non-invasive approaches to cancer treatment [5]. PDT operates through the use of photosensitizers that, when activated by specific wavelengths of light, produce reactive oxygen species (ROS), leading to the apoptosis of cancer cells [6, 7]. In a similar vein, SDT relies on ultrasound to stimulate sonosensitizers, thereby achieving a comparable cytotoxic effect [8, 9]. Nevertheless, the effectiveness of PDT is constrained by the limitations of light penetration in tissues and the availability of oxygen within those tissues. Conversely, while SDT offers the advantage of greater tissue penetration, it typically necessitates elevated concentrations of sensitizers, which introduces potential toxicity concerns [7, 10].

SPDT, which integrates the use of light and ultrasound, demonstrates increased effectiveness in preclinical research by optimizing the activation of both photosensitizers and sonosensitizers [11, 12]. This combined methodology seeks to address the shortcomings associated with single-modal therapies, resulting in augmented reactive oxygen species (ROS) generation and an expanded therapeutic window [13, 14].

Nano-TiO₂ and phthalocyanines (Pcs) have garnered significant interest in the realm of cancer treatment, attributed to their distinctive physicochemical characteristics. Thanks to its capacity to produce reactive oxygen species (ROS) when exposed to light, nano-TiO₂ stands out as a suitable agent for photodynamic therapy (PDT) applications [15, 16]. Moreover, it possesses the capability to augment cell membrane permeability when subjected to ultrasound irradiation, positioning it as a potential facilitator for SDT [17]. The integration of precious metals like platinum (Pt) with TiO2 has shown to enhance the quantum yield of sonosensitizers, thereby improving the efficacy of SDT. For instance, Pt-TiO2 heterostructures have been developed to enhance reactive oxygen species (ROS) production, which is crucial for effective cancer cell ablation [18, 19]. Similarly, gold-deposited TiO2 nanocomposites have demonstrated increased ROS generation, leading to significant tumor suppression [20]. Additionally, iron-doped TiO2 nanodots have been shown to enhance ROS generation and offer Fenton-catalytic functions, further improving SDT efficacy (4). Fe3O4@TiO2 nanoparticles have been used to deliver doxorubicin, a chemotherapeutic agent, alongside SDT, resulting in a synergistic effect that enhances therapeutic efficacy and reduces side effects [21]. Moreover, the combination of SDT with starvation therapy using TiO2@Pt/GOx platforms has shown promise in promoting systemic tumor suppression by modulating the tumor microenvironment [19]. Various methods such as metal or nonmetal doping and dye sensitization have been applied to enhance the visible-light sensitivity of TiO₂, and metal-phthalocyanine complexes, especially metal–organic macrocyclic dyes, have shown significant potential in enhancing the photocatalytic performance of TiO₂ due to their intense light absorption and superior chemical and photostability properties [22]. On the other hand, Pc, a synthetic photosensitizer, has been shown to exhibit robust absorbance and effective ROS generation, solidifying its role as a potent agent in PDT [23]. Water-soluble gallium phthalocyanine (GaPc) has been shown to effectively induce ROS formation and cell death in breast cancer cell lines, particularly when used in SPDT [24]. Methylene blue and aluminum phthalocyanine tetrasulfonate have demonstrated significant cytotoxic effects in prostate cancer cell lines, with SPDT showing more pronounced cell viability loss compared to PDT and SDT alone [25]. Indium phthalocyanine–mediated SPDT has been found to be more effective than PDT or SDT alone in reducing cell viability and inducing apoptosis in gastric cancer cell lines [26].

The present study undertakes an examination of the cytotoxic properties of nano-TiO₂, CuPc, and the combination of nano-TiO₂/Pc on HepG2 cells, analyzing these effects in isolation as well as in conjunction with sonodynamic therapy (SDT), photodynamic therapy (PDT), and sonophotodynamic therapy (SPDT). Our hypothesis posits that the synergistic application of these treatments, especially SPDT, will markedly augment cytotoxic effects through the mechanisms of oxidative stress induction and the activation of mitochondrial apoptotic signaling pathways.

## Materials and Methods

### Materials

Titanium (IV) isopropoxide (Ti[OCH(CH_3_)_2_]_4_/Ti(OPr)_4_), hydrochloric acid (HCl, 37%), and 1-propanol (CH_3_CH_2_CH_2_OH) were used for nano-TiO₂ synthesis. Copper (II) phthalocyanine (CuPc, dye content > 99%) was used for nanoparticle modification. All reagents were purchased from Merck. Lipid peroxidation (MDA), reduced glutathione (GSH), superoxide dismutase (SOD), and catalase activity assay kits were obtained from Baijin Co. Ltd. (Shanghai, China). Antibodies against caspase-8, cleaved caspase-8, caspase-9, cleaved caspase-9, caspase-3, cleaved caspase-3, Bax, Bcl-2, cyt-c, and β-actin, along with anti-mouse and anti-rabbit secondary antibodies, were purchased from Cell Signaling. The HepG2 cell line was obtained from ATCC. Cell culture reagents, including DMEM, FBS, streptomycin, penicillin, sodium bicarbonate, MTT, and DMSO, were sourced from Sigma Aldrich. All kits and reagents were used according to the manufacturers’ instructions.

### Synthesis of Pure TiO2 and Copper (II) Phthalocyanine–Modified TiO2 Nanoparticles

TiO₂ nanoparticles were synthesized by the sol–gel method in a reflux system. To synthesize pure TiO₂ nanoparticles, Ti[OCH(CH₃)₂]₄ and 1-propanol were weighed into a reaction vessel and a mixture of HCl, and water was added to this solution. For the synthesis of copper (II) phthalocyanine–modified TiO_2_ nanoparticles (CuPc-TiO_2_), CuPc dissolved in 1-propanol was added to the solution containing Ti[OCH(CH₃)₂]₄, HCl, and water and magnetically stirred [27]. After stirring, a clear, homogeneous solution was obtained. The molar ratios of Ti[OCH(CH₃)₂]₄:HCl:1-propanol:H₂O were 10:4.8:0.002:2.6. The solution was taken into a reflux system, and the reaction was carried out in an oil bath at 90 °C for 5 h. The resulting powders were separated by centrifugation [28].

### Cell Culture

Human hepatocellular carcinoma (HepG2) cells were cultured in DMEM supplemented with 10% heat-inactivated FBS, 1% streptomycin-penicillin, and sodium bicarbonate. Cells were maintained at 37 °C in a humidified incubator with 5% CO_2_ atmosphere and passaged at approximately 80% confluence [29].

### Experimental Design

This study was designed to investigate the cytotoxic and apoptotic effects of nano-TiO₂, copper (II) phthalocyanine (CuPc), and their conjugated form (nano-TiO₂/Pc) on HepG2 cells under different treatment modalities. For this purpose, HepG2 cells were initially divided into four main groups:

1. Control group: No treatment was applied.
2. Nano-TiO₂ group: Cells were treated with titanium dioxide nanoparticles alone.
3. CuPc group: Cells were treated with Copper (II) phthalocyanine alone.
4. Nano-TiO₂/Pc group: Cells were treated with a combination of nano-TiO₂ and CuPc, forming a nanocomplex.

Each of these treatment groups was further divided into three subgroups based on the type of activation applied:

- SDT (sonodynamic therapy) Treated with ultrasound exposure only.
- PDT (photodynamic therapy) Treated with light exposure only.
- SPDT (sonophotodynamic therapy) Treated with both ultrasound and light exposure simultaneously.

This experimental design allowed for the comparative analysis of the individual and combined effects of the compounds and activation methods on cell viability, oxidative stress levels, and apoptosis-related parameters.

### Effective Doses

HepG2 cells seeded in 48-well cell culture plates (1 × 10^5^ cells/mL) were incubated under cell culture conditions for 24 h. After completion of the incubation period, the adherent cells were incubated with different concentrations of nano-TiO₂ (0–50 µM), CuPc (0–50 µM), and nano-TiO₂/Pc (0–50 µM) prepared in fresh medium for another 48 h. Concurrently, cells were exposed to ultrasound (0–0.05 W/cm^2^, 1 MHz) and light (0–2 mJ/cm^2^, 60 s). Cytotoxicity was assessed using the MTT assay.

### Application of Sonodynamic, Photodynamic, and Sonophotodynamic Therapies

The cells were seeded at a density of 1 × 10^5^ cells/mL in both 24-well and 6-well plates and incubated for 24 h. After 4-h incubation with 48-h IC50 values of nano-TiO_2_ (10 µM), CuPc (13 µM) or nano-TiO_2_/Pc (6 µM), the following procedures were applied: 

- **SDT**: The cells were exposed to ultrasound at 1.0 MHz and 0.5 W/cm^2^ for 60 s.
- **PDT**: The cells were exposed to light at an intensity of 2 mJ/cm^2^ for 60 s (power density is approximately 0.033 mW/cm^2^).
- **SPDT**: The cells were exposed to light (2 mJ/cm^2^ for 60 s (0.033 mW/cm^2^)). Thirty minutes after ultrasound treatment (1.0 MHz, 0.5 W/cm^2^).

After treatments, the medium was renewed, and the cells were incubated for another 24 h. Cell viability in 24-well plates was assessed using the MTT assay, while cells in 6-well plates were used for apoptosis and oxidative stress evaluations. Furthermore, the Combination Index (CI) was calculated in order to ascertain the synergism effects. The interaction between SDT-TiO₂/Pc and PDT-TiO₂/Pc was evaluated by means of the Chou-Talalay method, whereby CI is expressed as follows: CI = (D)1/(DX)1 + (D)2/(DX)2 + (D)1 [30, 31].

### Evaluation of Apoptotic Processes

Proteins were separated by sodium dodecyl sulfate–polyacrylamide gel electrophoresis (SDS-PAGE, 8–12%) and transferred to polyvinylidene difluoride (PVDF) membranes using the methods described by Aksel (2021) in our previous study. Subsequently, the samples were then shaken overnight at 4 °C with primary antibodies prepared in 3% BSA, including caspase-3 (1:500), Bax (1:1000), Bcl-2 (1,1000), caspase-8 (1:500), cleaved caspase-3 (1:500), cleaved caspase-8 (1:500), cleaved caspase-9 (1:500), cyt-c (1:1000), and caspase-9 (1:500). The washed membranes were then incubated for 2 h with the respective secondary antibodies (1:2000–1:3000) prepared in 3% BSA. The β-actin antibody was used for normalization. Enhanced chemiluminescence (ECL) was used to visualize the bands, which were digitized with the Syngene GBOX system. The intensities of the bands were then calculated using ImageJ software [32].

### Markers of Oxidative Stress Assessment

Oxidative stress was evaluated by measuring SOD, CAT, and GSH activities, and MDA levels using commercially available ELISA kits, following the manufacturers’ instructions.

### Statistical Analysis

Analysis results are presented as mean ± standard deviation (*n* = 3). Statistical significance was determined using one-way ANOVA followed by Tukey’s post hoc test. *p*-value < 0.05 was considered statistically significant. GraphPad Prism (GraphPad Software, Inc., La Jolla, CA, USA) was used for statistical analysis and data visualization.

## Results

### Characterization of Pure TiO2 and Copper (II) Phthalocyanine–Modified TiO2 Nanoparticles

**Fig. 1:**
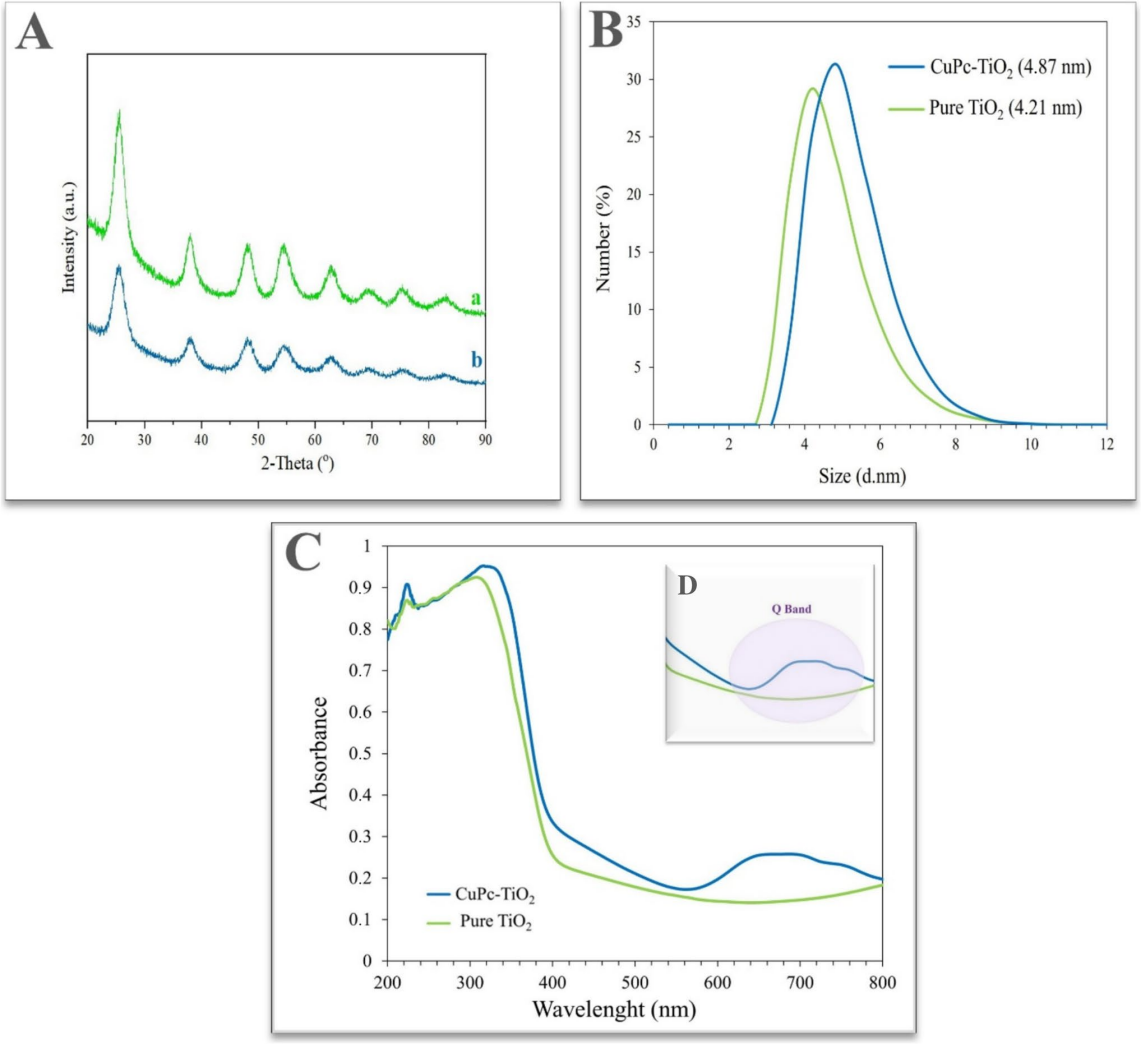
Characterization of pure TiO_2_ and copper (II) phthalocyanine–modified TiO2 nanoparticles. **A** XRD pattern of (a) pure TiO_2_ and (b) CuPc-TiO_2_ nanoparticles. **B** The size distribution of pure TiO_2_ and CuPc-TiO_2_ nanoparticles by number. **C** UV–Vis absorbance spectra of pure TiO_2_ and CuPc-TiO_2_ nanoparticles. **D** The characteristic Q band of CuPc-TiO₂ in the range of 650–700 nm

The X-ray diffraction (XRD) patterns of pure TiO_2_ and CuPc-TiO_2_ are shown in Fig. 1A. The sharp and well-defined peaks confirm the anatase phase in both samples. The peaks at 25.4°, 37.9°, 48.2°, 53.9°, 63.4°, 68.8°, and 75.2° 2θ correspond to the (101), (004), (200), (211), (204), (220), and (301) planes of anatase TiO_2_ (reference card no. 00–021–1272). The narrower peaks in pure TiO2 indicate higher crystallinity compared to CuPc-TiO_2_. The incorporation of CuPc slightly alters peak intensity and width, but the anatase phase is preserved [33].

Particle size analysis revealed average sizes of 4.21 nm for pure TiO_2_ and 4.87 nm for CuPc-TiO_2_. The slight increase in size for CuPc-TiO_2_ is attributed to CuPc addition, indicating successful surface modification (Fig. 1B). Both samples showed narrow size distributions, confirming uniform particle sizes [34].

UV–Vis absorbance spectra (Fig. 1C) showed that pure TiO_2_ absorbs strongly in the UV region (< 400 nm), while CuPc-TiO_2_ exhibits extended absorbance into the visible region (600–800 nm) due to CuPc incorporation. The red shift and broadening of the absorbance spectrum in CuPc-TiO_2_ suggest enhanced visible-light harvesting capabilities, making it suitable for photocatalysis [35]. The Q band in the 650–700 nm range confirms the presence of metallophthalocyanine in the sample CuPc-TiO_2_ sample (Fig. 1C).

### Cytotoxicity of Nano-TiO_2_, CuPc, and Nano-TiO_2_-Modified Phthalocyanine

**Fig. 2:**
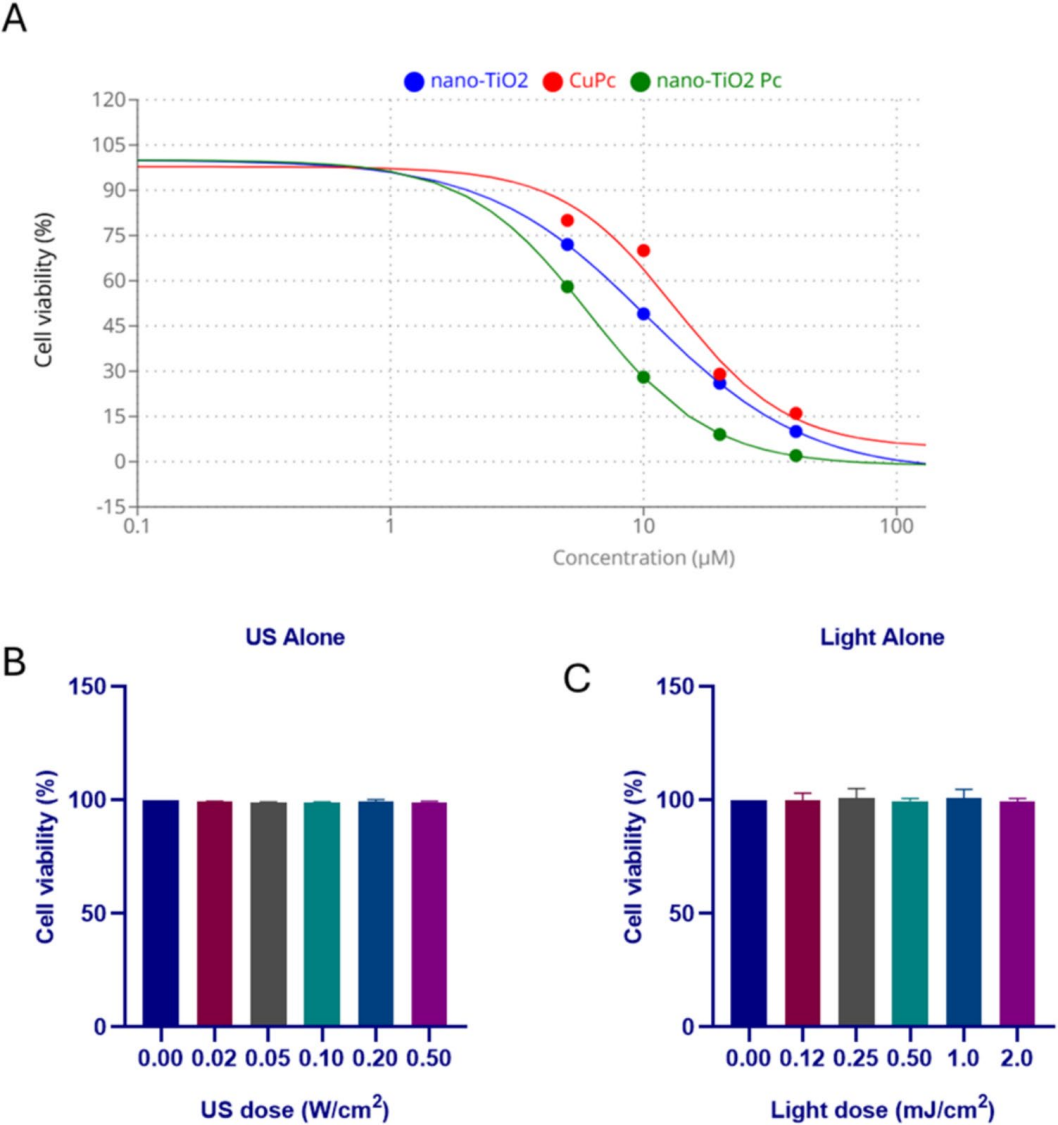
**A** Effects of various treatments on HepG2 cell viability after 48 h. IC50 values for nano-TiO_2_, CuPc, and nano-TiO_2_/Pc. **B** Effect of different light doses (alone) on cell viability for 24 h. **C** Effect of different ultrasound doses (alone) on cell viability for 24 h. Data are presented as mean ± SD (*n* = 3)

The cytotoxic effects of nano-TiO₂, CuPc, and nano-TiO₂/Pc on HepG2 cells were evaluated by MTT assay at the end of 24-h incubation period. IC50 values were determined as 10 µM, 13 µM, and 6 µM for nano-TiO₂, CuPc, and nano-TiO₂/Pc, respectively (Fig. 2A). Notably, the nano-TiO₂/Pc combination exhibited a lower IC50 value, suggesting enhanced antiproliferative activity compared to individual agents. Light alone and ultrasound alone treatments did not significantly affect cell viability, with different doses showing no toxicity (Fig. 2B, C).

### Effects of Treatments on HepG2 Cell Viability

**Fig. 3:**
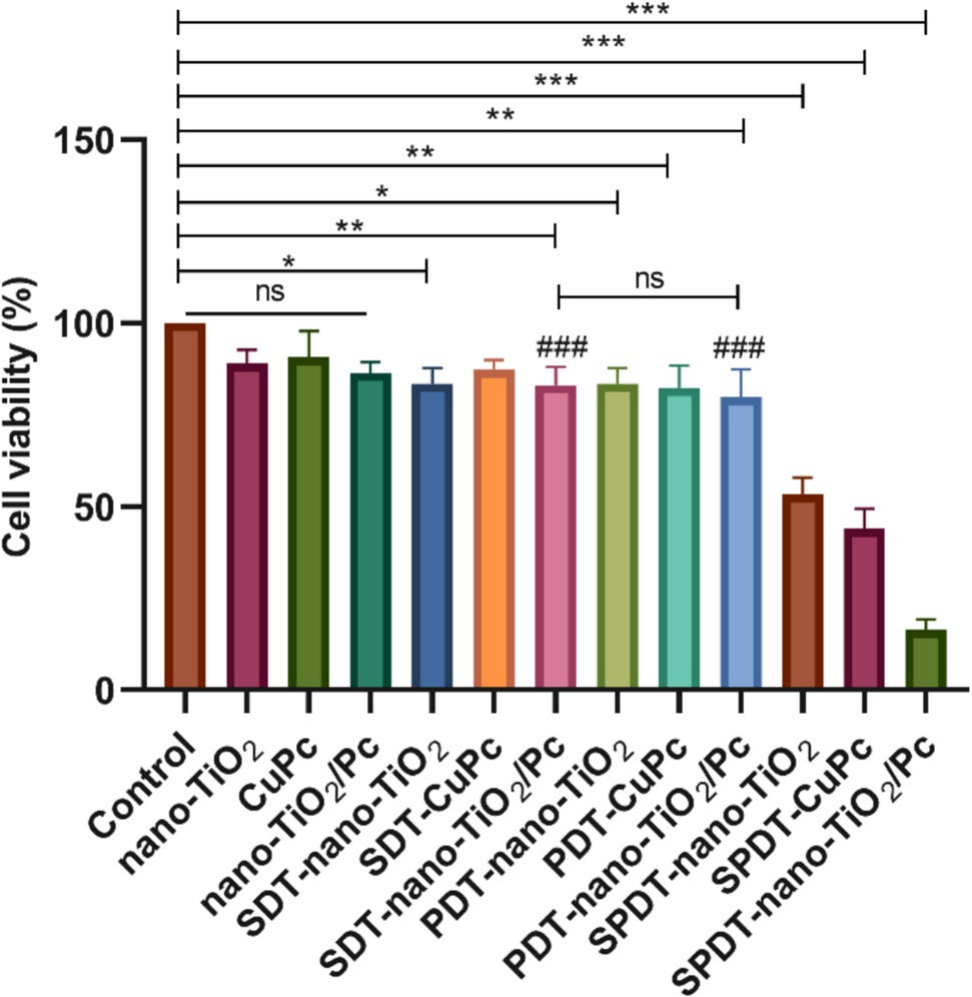
Effects of various treatments on HepG2 cell viability after 24 h. Cell viability was assessed using the MTT assay and presented as a percentage compared to the untreated control group. Data are presented as mean ± SD (*n* = 3). *p < 0.05, **p < 0.01, and ***p < 0.001 indicate statistical significance. ^#^*p* < 0.05, ^##^*p* < 0.01, and ^###^*p* < 0.001 compared to SPDT-TiO_2_/Pc group. “ns” indicates no statistically significant difference

MTT assays were performed to evaluate the effects of SDT, PDT, and SPDT on HepG2 cell viability. As shown in Fig. 3, individual treatments with nano-TiO₂ (10 µM) or CuPc (13 µM) did not significantly reduce cell viability. Similarly, the combination of nano-TiO₂/Pc (6 µM) alone had no significant effect. However, combining these treatments with SDT or PDT led to decreased cell viability. In particular, SDT-nano-TiO₂ (*p* < 0.05), PDT-nano-TiO₂ (*p* < 0.05), SDT-nano-TiO₂/Pc (16.89%, *p* < 0.01), and PDT-nano-TiO₂/Pc (20.22%, *p* < 0.01) significantly reduced cell viability compared to the control group. It was observed that SPDT-nano-TiO2/Pc and SPDT-nano-TiO₂ (*p* < 0.001) and SPDT-CuPc (*p* < 0.001) groups exhibited significant cell viability decreases. SPDT-nano-TiO₂/Pc group exhibited the most significant effect on cell death with a rate of 83.80% (*p* < 0.001 compared to control). Although the PDT-nano-TiO₂/Pc group (20.22%) exhibited slightly greater cytotoxicity compared to PDT-nano-TiO₂ (16.63%) and PDT-CuPc (17.7%), this increase was not statistically significant (*p* > 0.05). The doses required to achieve 83.80% apoptosis from SDT-nano-TiO2/Pc and PDT-nano-TiO2/Pc treatments were calculated separately (29.76 µM and 24.86 µM, respectively). The Chou-Talalay equation was applied, resulting in a calculated value of 0.49 for CI.

### Effects Treatments on the Expression of Mitochondrial Apoptotic Pathway Proteins in HepG2 Cells

To investigate the involvement of the mitochondrial apoptotic pathway in the observed treatment effects, Western blot analysis was performed to assess the expression levels of key proteins in this pathway after 24 h of treatment. The control group was left untreated (Fig. 6A).

**Fig. 4:**
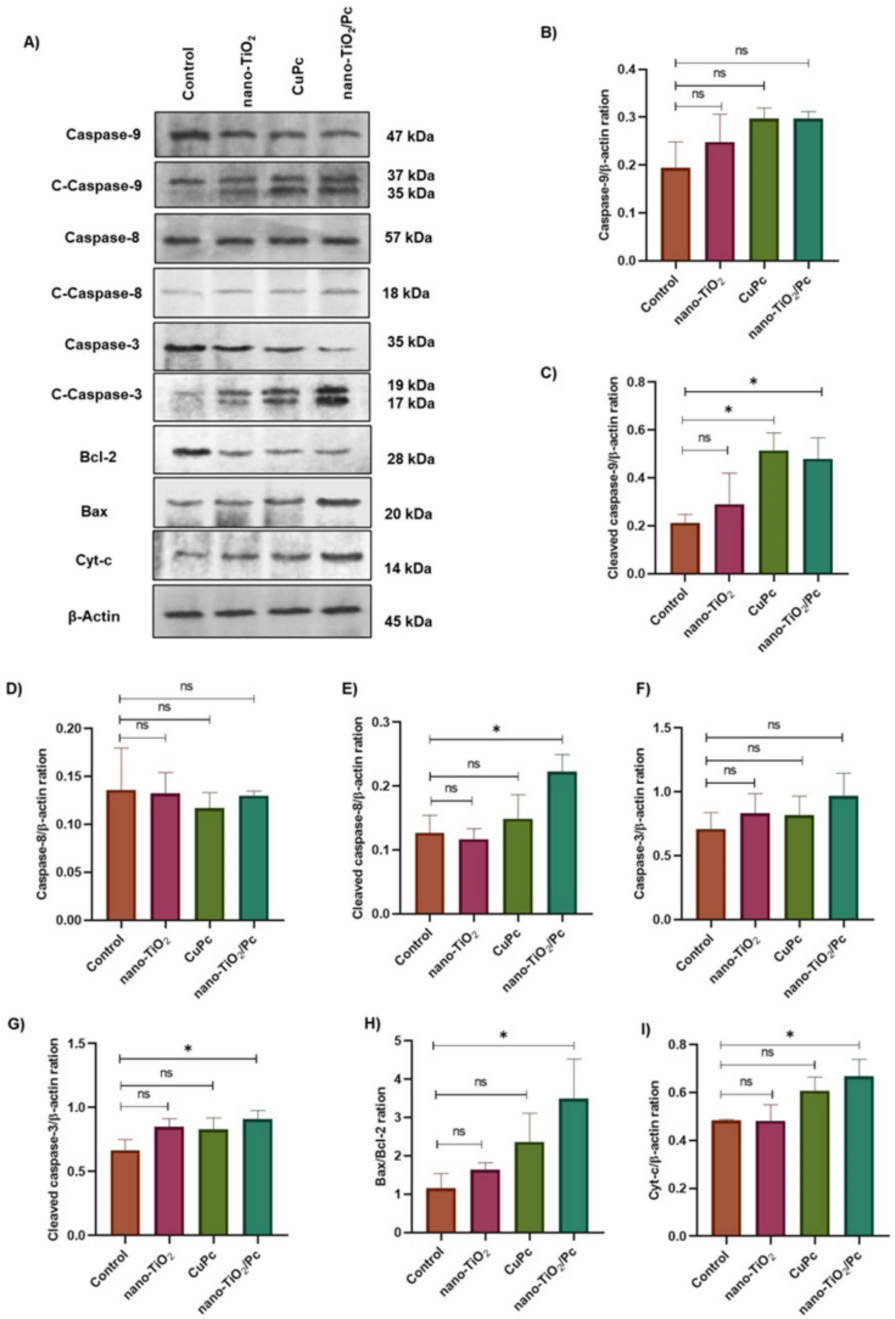
Western blot analysis of apoptosis-related protein expression in HepG2 cells. **A** Representative Western blot images showing the expression of caspase-9, cleaved caspase-9, caspase-8, cleaved caspase-8, caspase-3, cleaved caspase-3, Bcl-2, Bax, cytochrome-c, and β-actin in HepG2 cells treated with nano-TiO₂, CuPc, and nano-TiO₂/Pc. **B**–**I** Densitometric quantification of the protein bands normalized to β-actin. Data are presented as mean ± SD (*n* = 3). Statistical significance was determined using one-way ANOVA followed by Tukey’s post hoc test. *p < 0.05 was considered statistically significant. “ns” indicates no statistically significant difference

Treatment with nano-TiO_2_, CuPc, and nano-TiO_2_/Pc did not cause statistically significant changes in total caspase-9 expression in comparison with the control group, as demonstrated in Fig. 4A and measured in Fig. 4B. Similarly, no significant changes in cleaved caspase-9 levels were observed in the nano-TiO₂ group (Fig. 4A, C). However, CuPc and nano-TiO₂/Pc treatment led to a statistically significant increase in cleaved caspase-9 levels compared to the control (*p* < 0.05) group.

**Fig. 5:**
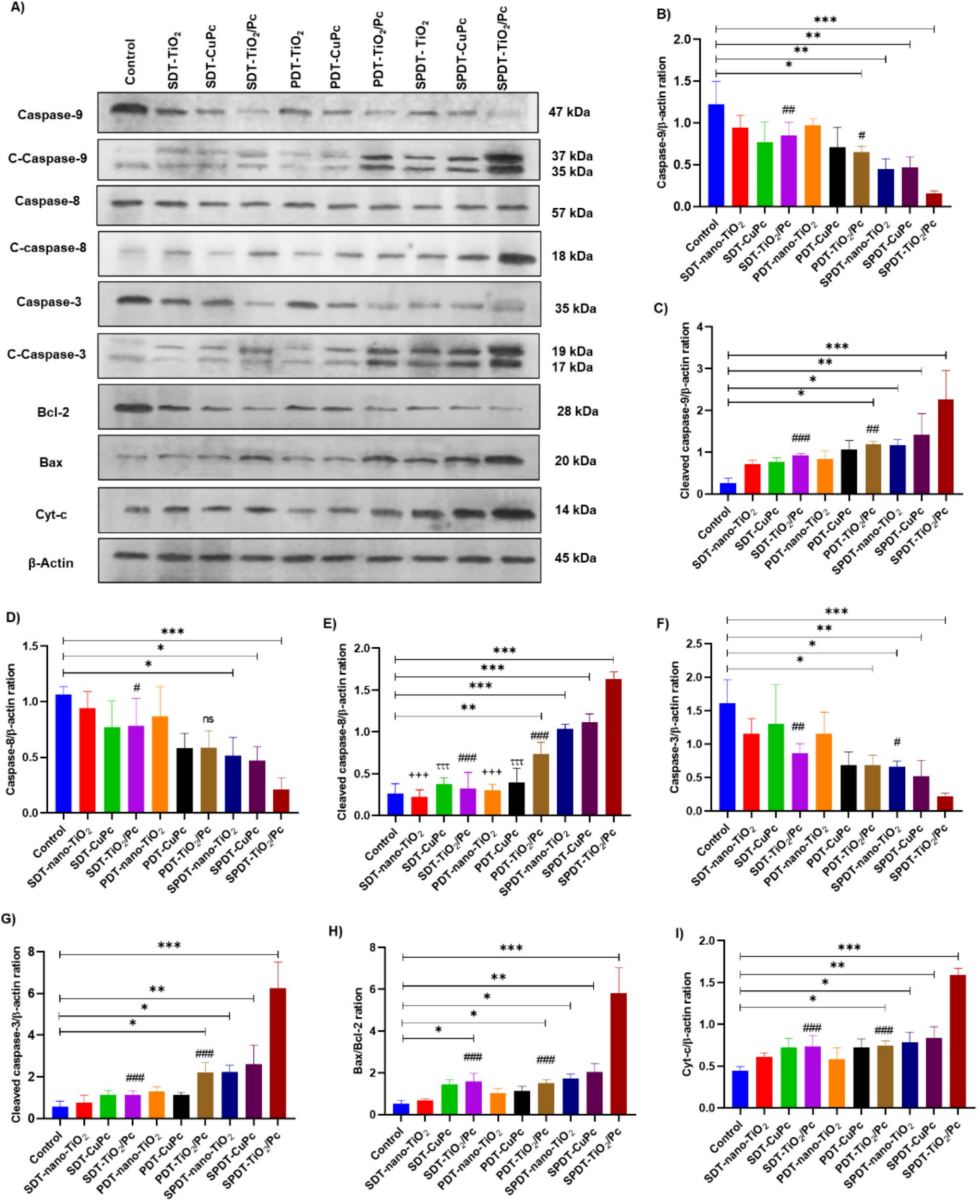
Western blot analysis of apoptosis-related proteins in HepG2 cells after various treatments. **A** Representative blots showing the expression of caspase-9, cleaved caspase-9, caspase-8, cleaved caspase-8, caspase-3, cleaved caspase-3, Bcl-2, Bax, cytochrome-c, and β-actin (loading control) in HepG2 cells subjected to different treatments: Control, SDT-nano-TiO₂, SDT-CuPc, SDT-TiO₂/Pc, PDT-nano-TiO₂, PDT-CuPc, PDT-TiO₂/Pc, SPDT-nano-TiO₂, SPDT-CuPc, and SPDT-TiO₂/Pc. Molecular weight markers are indicated in kDa. **B**–**I** Densitometric quantification of protein band intensities, normalized to β-actin. Data are presented as mean ± SD (*n* = 3). Statistical significance was determined using one-way ANOVA followed by Tukey's post hoc test. *p < 0.05, **p < 0.01, and ***p < 0.001 compared to control group; ^#^*p* < 0.05, ^##^*p* < 0.01, and ^####^*p* < 0.001 compared to SPDT-TiO2/Pc group; ^++++^*p* < 0.001 compared to SPDT-nano-TiO_2_ group; ^τττ^*p* < 0.001 compared to SPDT-CuPc group. “ns” indicates no statistically significant difference

Total caspase-9 levels generally decreased with SDT, PDT, and SPDT treatments, particularly in the SPDT-nano-TiO₂/Pc group (*p* < 0.001) (Fig. 5A, B). Cleaved caspase-9 levels increased significantly in the PDT-nano-TiO₂/Pc (*p* < 0.05), SPDT-nano-TiO₂ (*p* < 0.05), SPDT-CuPc (*p* < 0.01), and SPDT-nano-TiO₂/Pc (*p* < 0.001) groups compared to the control (Fig. 5A, C).

While no statistically significant change in total caspase-8 expression was observed in all treatment groups compared to the control group, the cleaved caspase-8 level was significantly upregulated in the nano-TiO_2_/Pc group (see Fig. 4A, D, E).

Total caspase-8 levels decreased across most treatments, with the most significant reduction in the SPDT-nano-TiO₂/Pc group (*p* < 0.001) (Fig. 5A, D). Cleaved caspase-8 levels increased significantly in the PDT-nano-TiO₂/Pc (*p* < 0.01), SPDT-nano-TiO₂ (*p* < 0.001), SPDT-CuPc (*p* < 0.001), and SPDT-nano-TiO₂/Pc (*p* < 0.001) groups (Fig. 5A, E).

As was the case with caspase-8 and −9, no statistically significant changes in the expression of total caspase-3 were observed in any of the treatment groups in comparison with the control group (Fig. 4A, F). However, nano-TiO₂/Pc treatment induced a statistically significant increase in cleaved caspase-3 levels compared to the control groups (*p* < 0.05) (Fig. 4A, G). Total caspase-3 levels decreased, especially in the SPDT-nano-TiO₂/Pc group (*p* < 0.001) (Fig. 5A, F). Cleaved caspase-3 levels increased significantly in the PDT-nano-TiO₂/Pc (*p* < 0.05), SPDT-nano-TiO₂ (*p* < 0.05), SPDT-CuPc (*p* < 0.01), and SPDT-nano-TiO₂/Pc (*p* < 0.001) groups (Fig. 5A, G).

Treatment with nano-TiO_2_/Pc resulted in a decrease in the expression of the anti-apoptotic protein Bcl-2 in comparison to the control group, while concomitantly resulting in an increase in the expression of the pro-apoptotic protein Bax (Fig. 4A). A quantitative analysis of the Bax/Bcl-2 ratio, a key indicator of apoptotic predisposition, revealed a statistically significant increase in the nano-TiO_2_/Pc group compared to the control group (*p* < 0.05). However, no statistically significant changes were observed in the nano-TiO_2_ and CuPc groups (Fig. 4H). SPDT-nano-TiO_2_/Pc, SPDT-CuPc, SDT-nano-TiO_2_, PDT-nano-TiO_2_, and SDT-nano-TiO_2_/Pc treatments resulted in a decrease in Bcl-2 expression and an increase in Bax expression in comparison to the control (Fig. 5A). The Bax/Bcl-2 ratio exhibited a significant increase in the SPDT-nano-TiO₂/Pc group (*p* < 0.001), the SPDT-CuPc group (*p* < 0.01), the SPDT-nano-TiO₂ group (*p* < 0.05), the PDT-nano-TiO₂/Pc group (*p* < 0.05), and the SDT-nano-TiO₂/Pc group (*p* < 0.05) (Fig. 5H).

Treatment with nano-TiO_2_/Pc resulted in a statistically significant increase in the level of expression of cyt-c in comparison with the control group (*p* < 0.05). The remaining treatment groups did not demonstrate any substantial alterations in comparison to the control group (see Fig. 4A and I). Cyt-c levels increased significantly in the SPDT-nano-TiO₂/Pc (*p* < 0.001), SPDT-CuPc (*p* < 0.01), SPDT-nano-TiO₂ (*p* < 0.05), and PDT-nano-TiO₂/Pc (*p* < 0.05) groups compared to the control (Fig. 5A, I).

To evaluate the potential synergistic effect of SPDT with nano-TiO₂/Pc, the Western blot results of the SPDT-nano-TiO₂/Pc group were statistically compared to those of both the SDT-nano-TiO₂/Pc and PDT-nano-TiO₂/Pc groups. Quantitative analysis demonstrated that the SPDT-nano-TiO₂/Pc group exhibited significantly elevated cleaved caspase-9 (*p* < 0.001, *p* < 0.01), cleaved caspase-3 (*p* < 0.001), cleaved caspase-8 (*p* < 0.001), and cyt-c (*p* < 0.001) levels compared to both SDT-nano-TiO₂/Pc and PDT-nano-TiO₂/Pc groups (Fig. 5C, G, I). The statistical comparison further confirmed the synergistic effect of SPDT, as SPDT-nano-TiO₂/Pc induced higher pro-apoptotic protein expression than either single treatment alone. In addition, the SPDT-nano-TiO₂/Pc group demonstrated a significant decrease in the anti-apoptotic protein Bcl-2 and a concurrent increase in the pro-apoptotic protein Bax (Fig. 5H), resulting in a significantly elevated Bax/Bcl-2 ratio (*p* < 0.001). SPDT-nano-TiO₂/Pc group exhibited significantly elevated Bax/Bcl-2 ratio compared to both SDT-nano-TiO₂/Pc and PDT-nano-TiO₂/Pc groups (Fig. 5H, p < 0.001). These findings collectively indicate that SPDT with nano-TiO₂/Pc effectively activates the mitochondrial apoptotic pathway and exerts a synergistic pro-apoptotic effect compared to SDT and PDT monotherapies.

The level of cleaved caspase-8 in SPDT-nano-TiO_2_ treatment was statistically significant compared to SDT-nano-TiO_2_ (Fig. 5E, p < 0.001) and PDT-nano-TiO_2_ (Fig. 5E, p < 0.001) treatments. Similarly, the effect of SPDT-CuPc compared with SDT-CuPc (Fig. 5E, p < 0.001) and PDT-CuPc (Fig. 5E, p < 0.001) was statistically significant. However, no statistical significance was found in the comparison of other proteins involved in the apoptotic pathway with SPDT combination therapies (Fig. 5A–D, F–I, p > 0.05).

### Effects of Treatments on Oxidative Stress Markers in HepG2 Cells

**Fig. 6:**
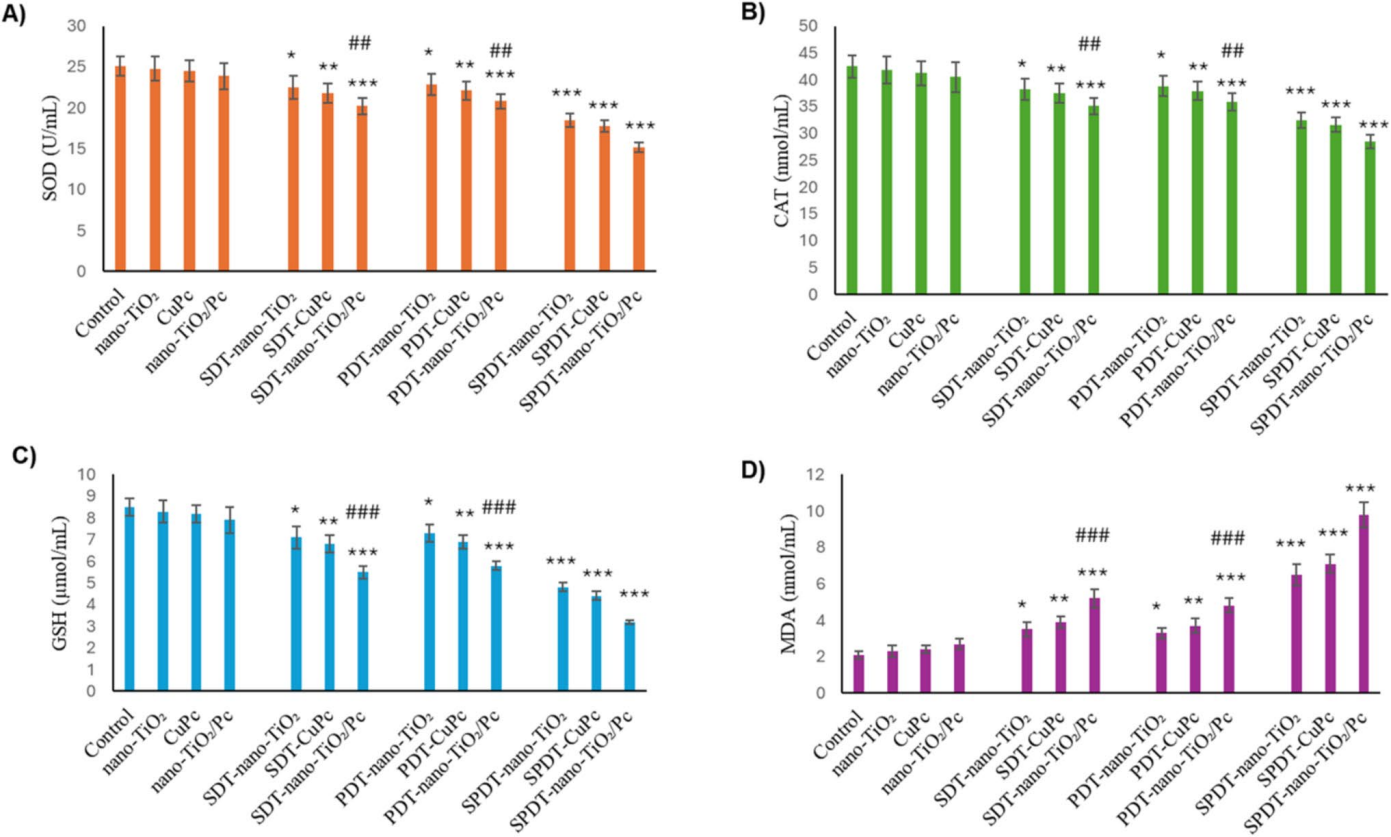
Effects of Different Treatments on SOD, CAT, GSH, and MDA Levels in HepG2 Cells. Statistical analyses were performed on data expressed as mean ± standard deviation (*n* = 3). Statistical significance was determined using one-way ANOVA followed by Tukey’s post hoc test. *p < 0.05, **p < 0.01, and ***p < 0.001 compared to the control group; ^#^*p* < 0.05, ^##^*p* < 0.01, and ^###^*p* < 0.001 compared to SPDT-TiO_2_/Pc group. SOD, superoxide dismutase; CAT, catalase; GSH, glutathione; MDA, malondialdehyde. SDT, sonodynamic therapy; PDT, photodynamic therapy; SPDT, sonophotodynamic therapy

Oxidative stress markers (SOD, CAT, GSH, and MDA) were measured using ELISA kits. The results are presented in Fig. 6. Individual treatments with nano-TiO₂, CuPc, or nano-TiO₂/Pc did not significantly alter these markers compared to the control group. However, combinations of SDT, PDT, or SPDT significantly decreased SOD, CAT, and GSH levels while increasing MDA levels (*p* < 0.05, *p* < 0.01, *p* < 0.001). The most pronounced responses were observed in SPDT treatments. In particular, the greatest oxidative stress was observed in SPDT-nano-TiO₂/Pc treatment (Fig. 6). To evaluate the efficacy of the combined treatment, the SPDT combination of TiO2/Pc treatment was compared with both SDT and PDT combination. A significant decrease in SOD (Fig. 6A, p < 0.01), CAT (Fig. 6B, p < 0.01), and GSH (Fig. 6C, p < 0.001) levels was observed when SPDT-nano-TiO₂/Pc treatment was compared with SDT-nano-TiO₂/Pc and PDT-nano-TiO₂/Pc treatments. MDA level was significantly lower in SDT-nano-TiO₂/Pc and PDT-nano-TiO₂/Pc treatments compared to SPDT-nano-TiO₂/Pc (Fig. 6C, p < 0.001).

## Discussion

This study aimed to evaluate the potential of combining nano-TiO₂, CuPc, and a nano-TiO₂/Pc with SDT, PDT, and SPDT for the treatment of HCC using HepG2 cells. The results demonstrate that SPDT, particularly when combined with nano-TiO₂/Pc, significantly enhances cytotoxicity, induces apoptosis, and increases oxidative stress in HepG2 cells compared to individual treatments or the agents alone.

The observed lack of significant cytotoxicity when nano-TiO₂, CuPc, or nano-TiO₂/Pc were applied individually (Fig. 3) is consistent with previous studies demonstrating that these agents often require activation by light or ultrasound to exert their full cytotoxic potential. For instance, Li et al. (2020) and McEwan et al. (2018) highlighted the necessity of light activation for photosensitizers in PDT to generate cytotoxic ROS [36, 37]. Similarly, the principle of SDT relies on ultrasound-mediated activation of sonosensitizers to produce ROS, leading to cell damage [38]. In our study, although the PDT-nano-TiO₂/Pc group showed slightly higher cytotoxicity (20.22%) compared to PDT-nano-TiO₂ (16.63%) and PDT-CuPc (17.7%), this increase was not statistically significant (*p* > 0.05). This limited response can be attributed to the inherent limitations of PDT when used alone. The efficacy of PDT is strongly influenced by factors such as light penetration, intracellular localization of the photosensitizer, and oxygen availability [39]. In the case of the nano-TiO₂/Pc complex, although both components are individually photoactive, their combined effect under light exposure may not yield a significantly enhanced therapeutic result. This could be due to potential barriers in intracellular distribution or limited activation under the specific PDT parameters used. In addition, the conjugation of nano-TiO₂ to the CuPc surface may have negatively affected cellular uptake by altering the molecular size and surface properties [40]. These structural changes could potentially worsen cellular uptake during light exposure. The minimal effect on cell viability observed in our study in combination of individual treatments and SDT alone or PDT alone emphasizes the importance of combining these agents with SPDT to achieve significant therapeutic effects.

The enhanced cytotoxicity observed with the combination treatments, especially SPDT, can be attributed to the synergistic effects of integrating ultrasound and light irradiation. The CI < 1 according to the Chou-Talalay method clearly demonstrates that SPDT-nano-TiO₂/Pc treatment exerts a synergistic cytotoxic effect on HepG2 cells. This quantitative validation further supports our conclusion regarding the increased efficacy of the combined treatment approach. US, as used in SDT, is known to induce cavitation, which can increase cell membrane permeability and potentially enhance the uptake of the sensitizer into cancer cells [38, 41]. This phenomenon is further supported by the work of Gong, Z. (2021), who emphasized the role of cavitation in enhancing the efficacy of SDT [42]. Both ultrasound and light can activate the sensitizers, leading to ROS production through different pathways. The use of nano-TiO₂/Pc in this study likely amplifies ROS generation, as TiO₂ nanoparticles are known to act as both photosensitizers and sonosensitizers, generating ROS under light and ultrasound irradiation, respectively [43].

The synergistic effect of combining SDT and PDT in SPDT is particularly noteworthy. SPDT integrates the advantages of both modalities, offering improved tissue penetration due to ultrasound and enhanced ROS production through the activation of both photosensitizers and sonosensitizers. This combined approach has been shown to overcome limitations associated with single-modality treatments, such as limited light penetration in PDT and the need for high sonosensitizer concentrations in SDT [44]. Our findings corroborate this, demonstrating that SPDT, especially when combined with nano-TiO₂/Pc, significantly enhances cytotoxicity compared to SDT or PDT alone.

The mechanistic underpinnings of the observed cytotoxicity involve the activation of both intrinsic and extrinsic apoptotic pathways, as evidenced by the alterations in apoptosis-related proteins. The significant increase in cleaved caspase-9, cleaved caspase-8, and cleaved caspase-3 levels, coupled with the elevated Bax/Bcl-2 ratio and cyt-c release, indicates that both mitochondrial and death receptor–mediated pathways are involved in the apoptotic process (Fig. 6). These results are consistent with previous studies showing that SDT and PDT can trigger apoptosis through mitochondrial pathways, involving caspase activation and cyt-c release [45, 46]. The marked activation of these apoptotic markers, particularly in the SPDT-nano-TiO₂/Pc group, underscores the potent pro-apoptotic effect of this combined treatment.

Oxidative stress plays a pivotal role in mediating the cytotoxic effects observed in this study. The significant decrease in SOD, CAT, and GSH levels, along with the increase in MDA levels, demonstrates that the combined treatments induce substantial oxidative stress in HepG2 cells (Fig. 6). This is consistent with the established understanding that both PDT and SDT exert their anticancer effects, in part, through ROS generation, leading to lipid peroxidation, DNA damage, and, ultimately, cell death [47, 48]. The particularly pronounced oxidative stress observed in the SPDT-nano-TiO₂/Pc group can be attributed to the enhanced ROS production resulting from the synergistic combination of SDT and PDT, along with the unique properties of nano-TiO₂/Pc. Similar findings have been reported in studies utilizing combined SDT and PDT approaches, where enhanced ROS generation and oxidative stress were key factors contributing to the observed cytotoxicity [49–51].

The study by Fazlı et al. on axially substituted silicon phthalocyanine-modified nano-TiO_2_ thin films and the work of Tekintas et al. on triazole-based Cu(II) and Zn(II) phthalocyanines–modified TiO_2_ nanoparticles provide further context to our findings, although they do not directly evaluate the combined SDT/PDT approach [27, 28]. These studies highlight the importance of material modifications in enhancing the photocatalytic and photodynamic properties of TiO_2_-based systems. Similarly, Ziental et al. (2020) demonstrated the cytotoxic effects of zinc phthalocyanine and TiO_2_ nanoparticles on cervical cancer cells, underscoring the potential of these agents in cancer therapy [52].

Furthermore, the work of Salama, B. et al. on the cytotoxic impacts of TiO_2_ nanoparticles on HepG2 cells and the study by Erdogan and Cevik on the effects of myricetin on metastasis and invasion in rapamycin-resistant HepG2 cells provide additional insights into potential therapeutic strategies for HCC [29, 53]. These studies emphasize the importance of exploring novel compounds and combination therapies to improve treatment outcomes for liver cancer. Another study demonstrated that titanium dioxide TiO_2_ nanoparticles triggered apoptosis in TM4 cells through mitochondrial pathways. Bcl-2, Bax, caspase 3, caspase 9, p53, and cyt-c proteins were found to play a pivotal role in this process [54].

The findings of this study have significant implications for the development of more effective treatments for HCC. The demonstrated efficacy of nano-TiO₂/Pc in a combined SPDT approach highlights the potential of this novel agent for targeted and potent cancer therapy. The synergistic effect of combining SDT and PDT, along with the enhanced efficacy of nano-TiO₂/Pc, warrants further investigation in in vivo models and, ultimately, in clinical trials.

Future studies should focus on in vivo validation by assessing the efficacy and safety of nano-TiO₂/Pc in animal models of HCC. Additionally, exploring alternative nanoparticle modifications or functionalization strategies, such as different ligands, surface coatings, or metal ions in the Pc complex, may enhance targeting and therapeutic efficacy. Combining SPDT with other treatment modalities, such as immunotherapy or chemotherapy, could offer synergistic therapeutic benefits and help overcome resistance mechanisms [55–57]. Further optimization of SPDT parameters, including ultrasound frequency, intensity, light wavelength, and dosage, is necessary to maximize efficacy and minimize adverse effects. Lastly, elucidating the molecular mechanisms underlying SPDT, particularly those related to ROS generation, apoptosis induction, and oxidative stress response, will provide deeper insights into the observed synergistic effects and aid in refining this promising therapeutic approach.

## Conclusion

In this study, TiO₂ nanoparticles were synthesized utilizing a reflux condensation method in conjunction with the derived copper phthalocyanine derivatives. The characterization of the synthesized nanoparticles revealed that both the TiO₂ and the nano-TiO₂/Pc exhibited an anatase crystal structure, which is known for its favorable photocatalytic properties. Furthermore, the average particle size distribution was determined to be 4.21 nm for the TiO₂ nanoparticles and 4.87 nm for the nano-TiO₂/Pc. These measurements suggest a controlled synthesis process, yielding nanoparticles with dimensions conducive to enhanced reactivity and application in various fields.

This study provides compelling evidence that the combination of SDT and PDT, enhanced by nano-TiO₂/Pc, represents a promising therapeutic strategy for HCC. The observed synergistic effects, mediated by increased ROS production, oxidative stress, and apoptosis induction, highlight the potential of this approach to overcome the limitations of conventional treatments. The significant reduction in cell viability, particularly with SPDT-nano-TiO₂/Pc, underscores the efficacy of this combined modality. While further research, including in vivo validation and mechanistic studies, is needed to fully realize its clinical potential, these findings pave the way for the development of more effective and targeted therapies for this challenging malignancy.

## Data Availability

No datasets were generated or analysed during the current study.
